# The effect of a brief mindfulness-based intervention on personal recovery in people with bipolar disorder: a randomized controlled trial (study protocol)

**DOI:** 10.1186/s12888-019-2242-0

**Published:** 2019-08-22

**Authors:** Sunny H. W. Chan, Samson Tse, K. F. Chung, C. H. Yu, Raymond C. K. Chung, Herman H. M. Lo

**Affiliations:** 1 Department of Rehabilitation Sciences, The Hong Kong Polytechnic University, Hung Hom, Hong Kong; 2 Department of Social Work and Social Administration, The University of Hong Kong, Pok Fu Lam, Hong Kong; 3 Department of Psychiatry, The University of Hong Kong, Pok Fu Lam, Hong Kong; 4 Department of Psychology, The Azusa Pacific University, Azusa, USA; 5 Department of Applied Social Sciences, The Hong Kong Polytechnic University, Hung Hom, Hong Kong

**Keywords:** Mindfulness-based intervention, Personal recovery, Bipolar disorder

## Abstract

**Background:**

With the advent of the recovery movement in mental health, a humanistic paradigm shift has occurred, placing the focus on *personal recovery* (i.e., hope, identity, and life meaning) instead of functional or clinical recovery only (i.e., symptom reduction or increases in physical function). Along the journey of recovery, people with bipolar disorder (BD) struggle to cope with recurring mood fluctuations between depression and mania. Mindfulness-based interventions (MBIs) have the potential to result in improvements in personal recovery outcomes. Thus, this protocol will evaluate the efficacy and mechanisms of a brief MBI for helping individuals with BD with their personal recovery. It is hypothesized that adults with BD randomly assigned to a brief MBI intervention will report greater improvements in personal recovery than those in a waiting list control condition. In addition, it is hypothesized that such benefits will be mediated by improvements in emotion awareness, emotion regulation, and illness acceptance. Moreover, the specific stage of BD is hypothesized to moderate the beneficial effects of the brief MBI, such that those in the early stage of BD will report more benefits regarding emotion awareness and emotion regulation, whereas those in the late stage of BD will report more advantages concerning illness acceptance.

**Method:**

One hundred and fifty-four adults with BD will be recruited from hospitals and community settings for this research project. This study will use a mixed methods design. A randomized-controlled trial will be conducted to compare a brief MBI (four sessions in total) group and a waiting list control group. Assessments will be made at baseline, after intervention, and at six-month follow-up. In addition, a qualitative and participatory research method called Photovoice will be employed to further understand the experiences of the participants who receive the brief MBI along their personal recovery journey.

**Discussion:**

If the study hypotheses are supported, the findings from this research project will provide empirical support for an alternative treatment. Moreover, by identifying the mechanisms of the beneficial effects of the brief MBI, the findings will highlight process variables that could be specifically targeted to make MBI treatment even more effective in this population.

**Trial registration:**

This study is registered with the Chinese Clinical Trial Registry (ChiCTR- 1900024658). Registered 20th July 2019.

## Background

### A paradigm shift to personal recovery

Individuals with BD experience varying degrees of recurring mood fluctuations. For many individuals with BD, the condition is long lasting, resulting in impaired clinical and functional recovery, as reflected by a high relapse rate and a chronic recurrent course [1]. Usually, improvement or “recovery” is operationalized as a reduction in psychiatric symptoms or a restoration of cognitive, social, and occupational functioning [2]. However, with the advent of the recovery movement, a humanistic paradigm shift is occurring which puts greater focus on *personal recovery* from chronic illness. This approach focuses on outcomes such as connectedness, hope, optimism, identity, meaning in life, and empowerment [3]. With these significant changes in recovery orientation, the ultimate goals of treatment have shifted to improvements in well-being and the achievement of valued life goals, rather than focusing on symptom reduction or improved function per se.

Although there is an increasing interest in the outcomes that are the focus of a personal recovery approach, the existing evidence-based psychosocial interventions do not effectively address these outcomes in individuals with BD. Specifically, the current adjunctive interventions for BD are usually based on a biopsychosocial diathesis-stress model and a focus on developing knowledge and skills [4]. The primary emphases of these interventions usually include psychoeducation in strategies for stress management, the identification of early signs of recurrence, the importance of keeping regular lifestyle habits, and medication adherence to decrease relapse [5]. Unfortunately, the notion of personal recovery has received little attention in the existing psychosocial interventions [6]. Indeed, very few attempts have been made to integrate recovery elements into present interventions (e.g. [7],). Further research is needed to address this significant recovery-oriented intervention gap, especially when taking the characteristics of BD into consideration.

### Difficulties encountered by individuals with BD during the recovery process

Goal striving or goal setting is important in the personal recovery process [3]. However, the encouragement of goal setting within the process might be compromised by the “amplified emotionality characteristics” of BD [8]. Goal pursuit could elicit emotional responses in people with BD. Specifically, ambitious or excessive striving to meet goals elicit a manic mood [9]. This, of course, is quite challenging for individuals with BD, especially when they have significant deficits in emotion regulation [10]. Thus, it would be worthwhile identifying ways to facilitate better mood regulation, which could then facilitate the recovery process as people with BD pursue valued goals.

In addition, during the recovery journey, it is not uncommon for some people to remain symptomatic and functionally impaired despite treatment. As a result, they may adopt a sick role which gradually but increasingly impairs their quality of life [11]. Such difficulties would be aggravated by problems with a sense of self or self-stigma [12]. The loss of hope and morale along the recovery journey can be further reflected by the lack of acceptance of the illness. Thus, interventions which facilitate an acceptance of the illness could have profound beneficial effects in this population [13].

### Mindfulness is a viable intervention for facilitating personal recovery in individuals with BD

Mindfulness practice is a viable approach for helping individuals face the challenges of BD in the recovery process. Mindfulness-based approaches are popular in the treatment of a variety of psychiatric disorders and represent a “third wave” of psychosocial interventions [14]. Mindfulness has been defined as “paying attention in a particular way: on purpose, in the present moment, and nonjudgmentally” [15]. Indeed, the essence of mindfulness practice aligns well with the recovery perspective in terms of the dimensions of awareness and acceptance [6]. More specifically, mindfulness is viewed as an approach to deliberately enhance awareness of experiences in the present moment and promote non-judgmental acceptance of experiences [16]. Typically, mindfulness training is usually conducted in a group format which can enhance freedom, belonging, and resonance among participants [17].

The emotion regulation mechanism of mindfulness [18] has been linked to the relevance and benefits of BD. The cultivation of “non-striving” ideas in mindfulness practice should help people with BD to cope with the paradoxical speculation of goal pursuits in the recovery process [15]. In fact, a recent study conducted by the PI of the present proposal found that diminishing the goal-striving approach can help to regulate positive affect among people with BD [19]. Mindfulness training allows people to shift their mindset in order to embrace the principle of “letting go” [20], which could be a remedy for the upsurge in goal striving across time. Moreover, an MBI always emphasizes “being” with both pleasant and unpleasant experiences and distancing oneself to observe and accept emotions rather than responding reactively [21]. Further trials are warranted to investigate the benefits of an MBI by making the most of its essence to regulate both manic and depressive moods.

In addition, the use of an MBI is thought to enhance coping with the distress associated with persistent symptoms and internalized stigma [14]. It has been found that an MBI can moderate the relationship between unavoidable distressing experiences and mental health outcomes and ultimately lead to the mitigation of the detrimental effects of the distress [22]. The notions of acceptance and observer stance, as highlighted in an MBI, are thus consistent with personal recovery focusing on life meaning despite ongoing symptoms and stigmatization [13].

### Theory of the mechanisms of MBI

On the basis of a review of the research literature, Baer [23] proposed five mechanisms that underlie mindfulness training. First, such training reduces excessive emotional reactivity; with mindfulness training, individuals learn to observe their experiences non-judgmentally as they arise. Second, mindfulness training produces cognitive changes that allow participants to understand that thoughts are not facts. Thus, a non-judgmental and decentred view of one’s thoughts is a key component of the training. Third, mindfulness results in improved self-management that promotes a range of skills for coping with maladaptive cognitions and emotional responses. Fourth, learning the skill of non-judgmental observation of autonomic arousal, racing thoughts, muscle tension, or other responses contributes to a more adaptive response to these events. The first four mechanisms are consistent with the Monitor and Acceptance Theory (MAT), as described by Lindsay and Creswell [24]. They also highlight that attention awareness and acceptance are the key components for improving emotion regulation. Finally, mindfulness also leads to a fundamental shift contributing to an acceptance of all experiences, with which participants learn to avoid attempting to change, escape, or avoid physical or emotional discomfort. In fact, the implication of changing one’s self-concept and self-perspective [18] is also highlighted as an important psychological mechanism of change in mindfulness training.

### Staging model of BD

On the other hand, the staging approach to BD can help in understanding the role of an MBI within the process of personal recovery. The staging model of BD is quite promising in terms of informing potential tailored treatments according to the progression of the illness at any particular point in time [25]. For example, individuals in the early stages of BD may likely benefit from formal psychological therapy such as psychoeducation or skills training [1], whereas acceptance-based interventions or the encouragement of social participation, despite disability, might be more relevant to the later stages of BD [25]. Research has shown that providing stage-sensitive interventions to people with BD should be a more promising approach in terms of matching the idiosyncratic experiences or needs of people with BD at different stages along the recovery journey.

### Research gaps in applying an MBI to BD

Although a number of studies have examined the beneficial effects of an MBI in BD populations, the outcomes that have been the focus of these studies have assessed clinical and/or functional recovery rather than personal recovery outcomes (e.g. [26, 27],). *The mismatch between the existing available treatments and the desires for a personal recovery from individuals living with BD lead to a significant intervention gap*. Moreover, existing clinical trials have ignored the important role of BD stage as a potential moderator [6]. The validity of the staging model usually depends on its linkage to clinical or functional recovery outcomes [28]. Using personal recovery outcomes is a research gap that has not been investigated with respect to BD stages so far. Therefore, incorporating the stages of BD in this research project could provide novel insights by discerning the stage-specific MBI along the recovery process. In addition, the research has yet to evaluate the specific mechanisms that explain the beneficial effects of MBI treatment. The extant research has also yet to evaluate the benefits of MBI treatment from the perspective of the participants [29]. Thus, further qualitative investigation is needed to better understand the experiences of research participants along the recovery journey.

Usually, the time commitment required for MBI treatment is significant; treatment often lasts for about 2 months [30]. To improve the feasibility and acceptability of mindfulness interventions, there has been an exponential increase in the development and published randomized controlled trials of brief MBIs over the past decade. The increasing popularity of the brief MBI approach is quite promising in terms of its practicability and significant effect size on reducing negative affectivity as comparing with active control groups [31]. For instance, evidence suggests that significant changes occur in the first 4 weeks of standard mindfulness programme [32] and that even brief mindfulness training can buffer affect reactivity [33] among general population. However, the mechanisms that underlie the efficacy of brief mindfulness interventions and their beneficial effects on recovery outcome domains among people with BD are still awaiting investigation.

## Methods/design

### Aims of the research project

Here, we propose a research programme that addresses all of the aforementioned knowledge gaps, including research that will enable us to understand the psychological mechanisms underlying recovery [7]. Specifically, the aims of the research project are as follows:

1. To examine the effectiveness of a brief MBI for enhancing personal recovery in a sample of individuals with BD relative to a waiting list control condition.
2. To evaluate the mediating roles of emotion awareness, emotion regulation, and illness acceptance as mechanisms underlying the beneficial effects of the brief MBI.
3. To investigate the moderating role of BD stage on the beneficial effects of the brief MBI.
4. To better understand the participants’ lived experience of mindfulness in relation to the personal recovery process.

**Fig. 1:**
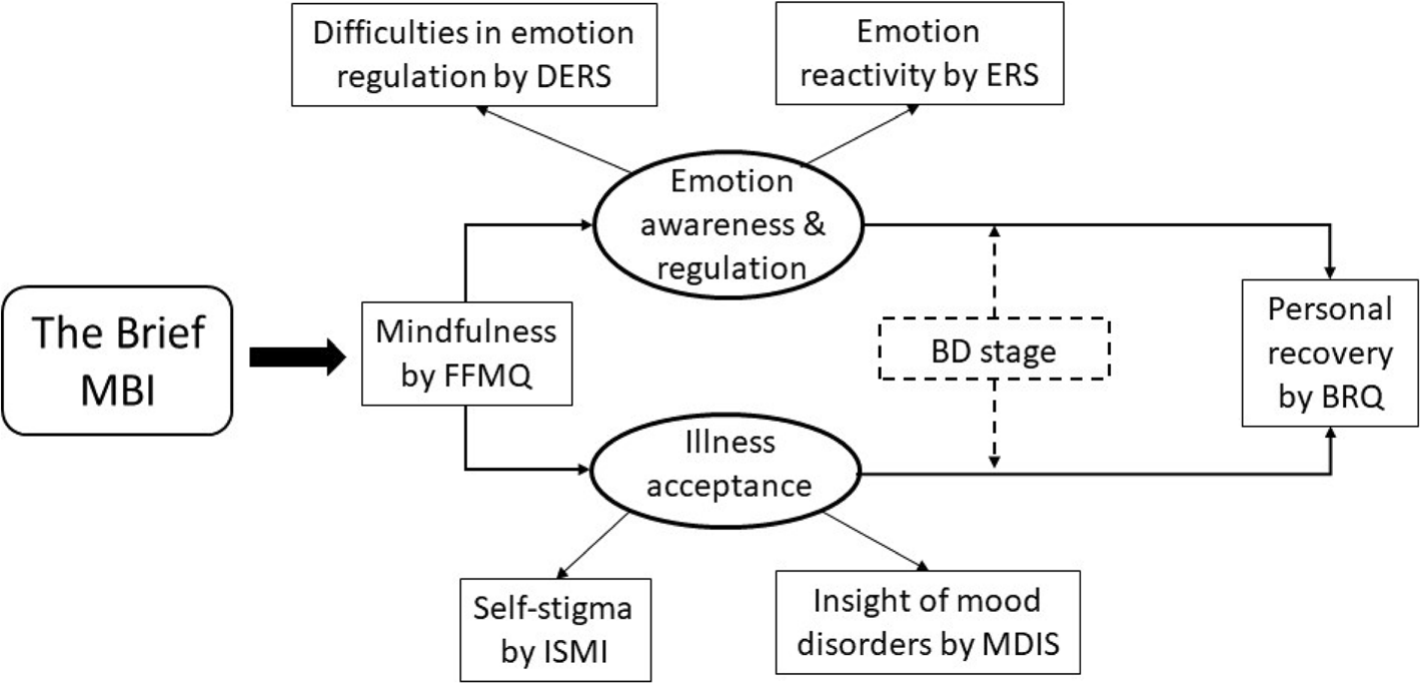
Conceptual model of potential mechanism of the brief MBI on personal recovery. Note: FFMQ = Five Facet Mindfulness Questionnaire; DERS = Difficulties in Emotion Regulation Scale; ERS = Emotion Reactivity Scale; ISMI = Internalized Stigma of Mental Illness; MDIS = Mood Disorders Insight Scale; BRQ = Bipolar Recovery Questionnaire

On the basis of the conceptual model depicted in Fig. 1, we will address these aims by testing the following specific hypotheses:

1. The brief MBI will be more effective, with a higher effect size, than the waiting list control on measure of the primary outcome of personal recovery.
2. The beneficial effects of the brief MBI on measure of personal recovery will be mediated by treatment-related increases in emotion awareness, emotion regulation, and illness acceptance.
3. The hypothesized mediation effects will be moderated by BD stage: that is, individuals with BD who report having a lower number of previous BD episodes will evidence stronger mediation effects for treatment-related changes in emotion awareness and emotion regulation, whereas individuals with BD who report having a higher number of previous BD episodes will evidence stronger mediation effects regarding illness acceptance.
4. The qualitative analysis will increase our understanding of the effects and mechanisms of the brief MBI interventions.

### Pilot studies

A pilot quantitative study was conducted by the first author in mid-2018. Twenty people with BD were recruited in a randomized controlled trial; half of them were assigned to a brief MBI group, while the other half were assigned to a waiting list control group. Comparisons were made of the two groups. Participants in the brief MBI group showed significant improvement on the measured variables. Specifically, the primary outcome of personal recovery had a moderate effect size (d = 0.35). Interestingly, there was virtually no effect of treatment on the secondary outcome of functional recovery (d = 0.02). Other potential mediators also showed small to moderate effect sizes (d = 0.22 to 0.38). The results of this study are summarized in Table 1. The feedback collected from participants was used to refine the programme content.

**Table 1:** Comparison of participants in brief MBI group and control group in a pilot study

Variable	Brief MBI group (*n* = 10)		Control group (n = 10)		Effect size
	Pre	Post	Pre	Post	
	Mean (sd)	Mean (sd)	Mean (sd)	Mean (sd)	Cohen’d
Independent variable (Mindfulness)					
FFMQ	117.4 (20.89)	122.3 (22.52)	116.7 (16.65)	114.9 (17.25)	0.37
Mediators (Emotion awareness and regulation)					
DERS	97.2 (22.68)	90.6 (19.89)	99.8 (17.43)	97.8 (18.07)	0.38
ERS	36.4 (24.78)	33.0 (22.83)	36.3 (12.95)	37.1 (13.0)	0.22
Mediators (Illness acceptance)					
ISMI	2.22 (.30)	2.1 (.34)	2.17 (.20)	2.18 (.19)	0.29
MDIS	11.15 (.97)	11.3 (.79)	11.2 (1.40)	11.0 (1.41)	0.26
Primary outcome (Personal recovery)					
BRQ	2327.5 (548.79)	2507.5 (504.43)	2322.5 (428.57)	2347.0 (394.73)	0.35
Secondary outcome (Functional recovery)					
FAST	27.3 (23.70)	26.6 (22.55)	27.3 (19.56)	27.1 (18.75)	0.02

*FFMQ* five facet mindfulness questionnaire, *DERS* difficulties in emotion regulation scale, *ERS* emotion reactivity scale, *ISMI* internalized stigma of mental illness, *MDIS* mood disorders insight scale, *BRQ* bipolar recovery questionnaire, *FAST* functional assessment short test

Further qualitative data were retrieved from another research project of the first author [8, 19]. Ninety people with BD in remission for at least 2 months were recruited and interviewed. They were asked to express their opinion on “staying well with BD along their recovery process”. Interview contents were transcribed and analysed by grounded theory. Three major themes were elicited [1]: Understanding BD: participants found that the sooner they were educated about and accepted their illness, the better chances they had of managing it [2]; Understanding my BD: self-awareness in the context of the illness demonstrated instrumental value in mood management and recovery, and learning ways to regulate emotions and counter stigma was found to be crucial in helping to facilitate “living well” with BD [3]; Setting up stay well plans: with knowledge of BD and what it meant to them, the participants became informed in terms of setting up stay well plans. All in all, *the participants expressed a strong need for a psychosocial intervention that would help them effectively manage their fluctuating moods and to find ways to live with the distress and difficulties of the disorder*. They also regarded a brief intervention to be more practical and efficient given their hectic living patterns. Such qualitative data informed the design of the brief MBI programme in the present proposal.

### Research design and procedure

This research will use a mixed methods design. First, a two-armed randomized-controlled trial will be employed to evaluate the clinical effectiveness of the brief MBI on personal recovery outcomes, with study participants randomized into a brief MBI group or a waiting list control group according to a computer-generated randomization list. The brief MBI will incorporate the basic theory and research of foundation mindfulness-based cognitive therapy (MBCT; 30) and is adapted from the treatment manual of MBCT for BD [21]. As usual, the programme will be conducted in group format [21, 30]. Furthermore, information and data gained from pilot quantitative and qualitative studies will be incorporated into the design and modification of the brief MBI intervention.

The brief MBI intervention will consist of four weekly sessions of 120 min each. The themes and contents of the brief MBI sessions are summarized in Table 2. The brief MBI will be conducted in groups of about 8 to 10 participants and led by therapists qualified in mindfulness teaching. Daily home practice will be encouraged and recorded for the participants in the brief MBI group. The waiting list control group will continue with routine care in hospital-based or community-based settings but will be offered the brief MBI intervention after 6 months (the final study follow-up assessment). While participants will be aware of the group to which they have been allocated, outcome assessors and data analysts will be kept blind to the allocation.

**Table 2:** Themes and contents of the brief MBI

Themes	Intention of interventions	Major mindfulness practice
1. Welcome in the moment	Introduction of mindfulness; experiential learning and practice of mindfulness; start off a mindfulness journey in daily life	➢ Movement exercise and brief sitting meditation practice ➢ Raisin exercises ➢ Yoga-Body scan ➢ Breathing space introduction ➢ Home practice
2. Responding with depression	Deepen mindfulness experience; experiential learning thoughts ➔ emotions relationship; emotion-focused meditation (depression); learning ways to respond with depression	➢ Body scan ➢ Mindful sitting with breath, sounds and thoughts ➢ Automatic negative thoughts scenarios (thoughts ➔ emotions) ➢ Emotion-focused meditation – depression ➢ Breathing space ➢ Home practice
3. Responding with mania	Deepen mindfulness experience; experiential learning emotions ➔ thoughts relationship; emotion-focused meditation (mania); learning ways to respond with mania	➢ Mindful walking ➢ Why be mindful with mania ➢ Automatic positive thoughts scenarios (emotions ➔ thoughts) ➢ Emotion-focused meditation – mania ➢ Breathing space acceptance ➢ Home practice
4. Self-care and Kindness to self	Deepen mindfulness experience; Cultivation loving-kindness and compassion to self	➢ Mindful sitting with breath, sounds and thoughts ➢ The compassionate coaching story ➢ Self-soothing activities with mindfulness ➢ Loving-kindness meditation ➢ Home practice

In addition, a supplementary qualitative research design using a participatory research methodology called photovoice [34] will be employed. This will help to facilitate the investigation of the in-depth mindfulness experience of participants during the brief MBI training as they go through the process of personal recovery. Through photo sharing and discussion, the participants will share their recovery-related perceptions and narratives [35]. Participants from the intervention group will be asked to take photos with their smartphones at the end of each session of the brief MBI programme. The themes of the photos will be about their lived experience of mindfulness in their recovery journey. They will also be asked to write up their reflections about the photos and invited to participate in a follow-up focus group interview, which will be held 1 month after the completion of the brief MBI programme. The format of the interview will be semi-structured, with a collaborative enquiry of the experiences gained from the programme.

**Fig. 2:**
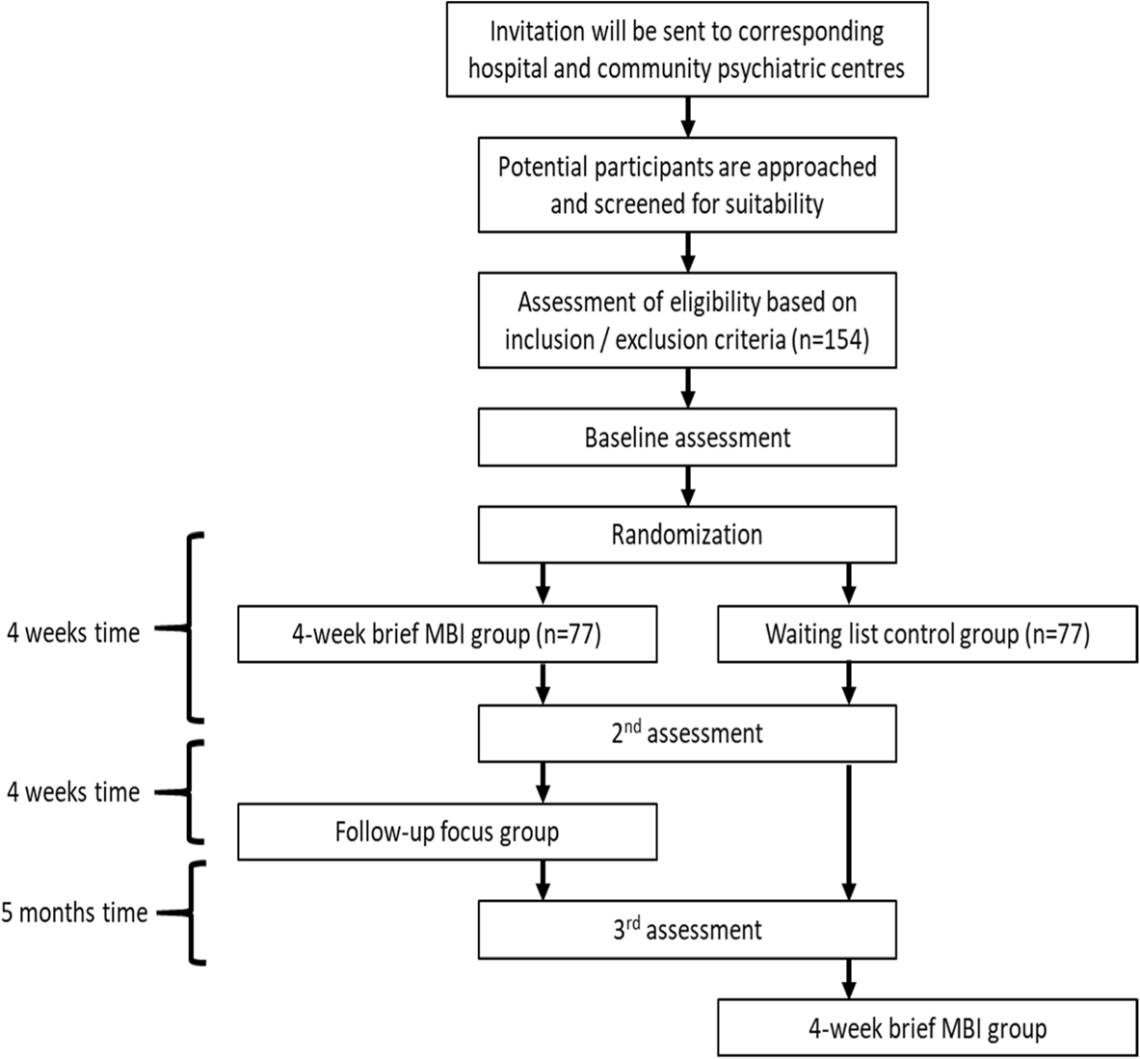
A flowchart of the recruitment and implementation

A flowchart of the recruitment and implementation of the whole research procedure is illustrated in Fig. 2. The protocol has adhered to the SPIRIT guidelines/methodology.

### Ethics

This study protocol has been approved by Human Subjects Ethics Sub-committee of the Hong Kong Polytechnic University (Reference number: HSEARS20190327010). An information sheet and a written consent form which has been formally approved by the ethics committee will be distributed to participants when they are invited to take part in the research project.

### Measurements

The psychometric attributes of all the measurement tools used in this project, such as reliability and validity, are psychometrically sound. For those assessments without a Chinese version, translation and cultural adaptation have been made with reference to the guidelines for the translation and adaptation of psychometric scales [36]. Outcome assessment will take place at baseline (T1), after intervention (T2), and at a six-month follow-up (T3). All participants will be assessed in terms of the following:

Primary outcome:

- ➢ Personal experiences of BD recovery: This will be assessed using the Bipolar Recovery Questionnaire (BRQ; 37). This 36-item questionnaire measures an individual’s experiences of recovery, such as personal understanding of self, sense of personal agency, or identifying recovery as a dynamic process. Total score is calculated by summing individual scores across all items. Evidence supports the internal consistency of the total score (*α* = .875) and good test-retest reliability over 4 weeks (*r* = .866) [37]. A higher total score indicates better personal recovery.
  Mediators:
- ➢ Emotion awareness and emotion regulation: These will be assessed using the Chinese version of the Difficulties in Emotion Regulation Scale (DERS [38];) and the Emotion Reactivity Scale (ERS [39];). The DERS is a 36-item questionnaire that measures adults’ difficulties in emotion regulation. Evidence supports the scale’s internal consistency (*α* = .9) and good test-retest reliability over 2 weeks (*r* = .84) [38]. A higher total score suggests greater problems with emotional regulation. The ERS is a 21-item self-report measure of emotion sensitivity, intensity and persistence. Evidence demonstrates the good consistency (*α* = .94) of the scale [39]. A higher total score indicates greater emotional reactivity.
- ➢ Illness acceptance: This will be assessed by the Internalized Stigma of Mental Illness Scale (ISMI [40];) and the Mood Disorders Insight Scale (MDIS [41];). The ISMI is a 9-item short form that measures self-stigma. Evidence supports the good consistency of the scale (*α* = .86) [40]. A higher total score reflects higher levels of reported internalized stigma. The MDIS is an 8-item scale that measures insight of mood disorders. Evidence supports the scale’s satisfactory test-retest reliability over 1 week (*r* = .75). Validity testing of the scale was based mainly on clinician ratings (r = 0.49) [41]. A higher total score indicates greater insight.
  Moderator:
- ➢ BD stage will be operationalized as the number of previous BD episodes [6, 25]. Consistent with previous prospective studies on BD stage [13, 42], the number of previous episodes will be categorized as less than 5, 5 to 10, and more than 10.
  Independent variable:
- ➢ Mindfulness: This will be assessed by the Chinese version of the Five Facet Mindfulness Questionnaire (FFMQ [43];). The FFMQ is a 39-item questionnaire which consists of five subscales: Observing, Describing, Acting with Awareness, Non-judging of Inner Experience, and Non-reactivity to Inner Experience. The five factors form a total mindfulness score which reflects a global measure of mindfulness. Evidence supports the scale’s good consistency (*α* = .83) and test-retest reliability over 2 weeks (*r* = .88) [43]. A higher total score indicates greater levels of mindfulness.

### Intervention fidelity

To ensure the fidelity of the intervention, qualified therapists who have basic professional training in mindfulness-based intervention (e.g., a one-year foundation course of teaching mindfulness-based cognitive therapy as organized by the Oxford Mindfulness Centre and the Hong Kong Centre for Mindfulness) plus at least 2 years’ experience in conducting mindfulness-based programmes will be invited to implement the brief MBI. Through a two-day training programme, a team of qualified therapists will learn the rationale and contents of the brief MBI. They will also develop and practice skills in conducting the brief MBI. All intervention sessions will be audiotaped and assessed by another team of independent raters who are also qualified therapists. They will ensure the intervention’s fidelity by following the mindfulness-based interventions teaching assessment criteria scale [44] and the designed intervention protocol. For the qualitative part of the research, the research team will receive a one-day training course to learn the corresponding interview questions and the process for conducting the focus groups. In addition, regarding the qualitative analysis, another team of independent raters will audiotape, transcribe, and review the contents of the qualitative interviews to identify themes.

### Sampling

On the basis of the data from the pilot study results and according to the G*Power programme [45], and in order to achieve a statistical power of 0.8 with a medium effect size (f = 0.35) and a significance level of 0.05 in repeated measures, a multivariate analysis of variance under the proposed two-group, three-time-point design and a total sample size of 82 will be needed. Furthermore, for the path model analysis of the underlying mechanism with two latent variables and seven observed variables, a sample size of 138 is needed, according to an online calculator [46]. In addition, and as recommended by Kline [47], the minimum sample size to parameters ratio should be 10:1 in a path model. As there will be 10 potential parameters in the proposed model, the minimum sample size needed is 100. Thus, we will plan for a total of 138 participants. Assuming an attrition rate of about 10%, a final sample size of 154 (i.e., 77 per condition) will be enrolled into the study and randomized.

The inclusion criteria are as follows: (1) a diagnosis of Bipolar I or II disorder; (2) in a state of full remission for more than 2 months [48]; (3) no active suicidal ideation or self-harm behaviours; (4) on regular medication; and (5) aged 18 years old or above. People with a comorbid diagnosis of schizophrenia, schizoaffective disorder, substance misuse, organic brain syndrome, or intellectual disability will be excluded. A Structured Clinical Interview for DSM-IV Disorders (SCID; only the mood disorders sections) [49] will be administered by trained clinicians to confirm the diagnosis and remission status of the participants. Observation of mental state and any potential for self-harm behaviours will be closely monitored throughout the intervention and assessment processes. When necessary, participants will be advised to consult a professional. After completing the project, participants will be given cash coupons as incentives. Suitable participants will be recruited from various hospital-based and community-based psychiatric rehabilitation settings across Hong Kong. With an agreed partnership with a local non-governmental organization (NGO) and a collaborated hospital, initial permission has been sought for recruiting participants from these settings.

### Data analysis

The data will be analyzed using SPSS, version 24.0. The associations among different variables will be investigated using Pearson’s correlation analysis. To test Hypothesis 1, a factorial ANOVA with repeated measures will be conducted. Hierarchical linear modelling (HLM) will be employed to test the individual growth model by studying repeatedly measured data. This integrated random-coefficients approach is usually conceptualized as a two-level model. Level 1, or the within-subject model, is the model for repeated measures with time-varying variables nested within individuals, whereas level 2, or the between-subject model, is the model for time-invariant variables between groups of individuals. SPSS mixed will be used in performing the HLM. In order to reduce the possible effects of multicollinearity, grand mean centring of different plausible predictors will be done before further data analysis. The fit of the models will be assessed and compared using appropriate fit statistics, including likelihood ratio tests and Akaike information criterion correction [50].

To test Hypotheses 2 and 3, the macro PROCESS SPSS and the bootstrapping approach [51] will be used to assess the moderated mediation effects in the path model. Each predictor will be mean centred to form the interaction terms. The effects of covariates such as age or gender will be adjusted accordingly. The PROCESS macro is based on ordinary least squares regression. In all analyses, the significance level will be set to *p* < 0.05.

To test Hypothesis 4, the contents of the focus group interview will be audiotaped and transcribed by a team of independent raters. Thematic analysis will be conducted with the help of the software NVivo (version 10.1.3) for storing and managing the data. The coding and analysis will use interpretative phenomenological analysis (IPA [52];). The IPA will allow a bottom-up approach to determine the themes. Throughout the analysis, the codes will be identified from frequently used words or ideas from the transcripts. Codes will then be categorized into larger recurrent themes for further analysis. The combined analysis of the Photovoice pieces and corresponding narratives will use the iconographic and thematic analyses approaches [53]. The research team will meet to discuss the significance of each theme and triangulate the results with the quantitative findings.

## Discussion

To facilitate the personal recovery process for individuals with BD, an appropriate personal recovery-oriented treatment is needed. Mindfulness-based interventions (MBIs) have the potential to address the existing research gap because its underlying elements can help to achieve the recovery outcomes. Specifically, the essences of awareness and acceptance that are central to an MBI could directly help individuals with BD to address the need for more emotion awareness and emotion regulation as well as illness acceptance. On the other hand, the long-time commitment in standard MBI programmes (which often lasts for about 2 months) limit the number of individuals willing to participate in such treatment. A brief MBI programme helps address the need for improving the feasibility and acceptability of mindfulness interventions. Moreover, considering the stage of the participant’s BD would be useful for better understanding how (and for which individuals with BD) a brief MBI would be most effective during the recovery process. To the best of our knowledge, using the personal recovery outcome is a research gap that has not yet been investigated with respect to BD stage. Moreover, the moderating influence of stage on mechanism and outcome variables has not yet been tested in a clinical trial examining the benefits of the brief MBI in individuals with BD.

In sum, it is hoped that this study can firstly address extant research and intervention gaps by evaluating the efficacy and mechanisms of a psychosocial intervention to facilitate the process of personal recovery in adults living with BD. Secondly, it can also provide a novel insight into the landscape in which the brief MBI is applied to a new research area of personal recovery among people with BD. Thirdly, this study can also integrate the staging model of BD with the beneficial effects of the brief MBI along the process of personal recovery. Fourthly, the qualitative analysis can help better understand the lived experience of mindfulness during the personal recovery process among individuals living with BD. Lastly, the important findings in this study can facilitate the development of our theoretical understanding of the change mechanisms important to the personal recovery process.

## Data Availability

Available upon request.

## Abbreviations

BD: Bipolar disorder
BRQ: Bipolar recovery questionnaire
DERS: Difficulties in emotion regulation scale
ERS: Emotion reactivity scale
FFMQ: Five facet mindfulness questionnaire
HLM: Hierarchical linear modelling
IPA: Interpretative phenomenological analysis
ISMI: Internalized stigma of mental illness scale
MBCT: Mindfulness-based cognitive therapy
MBI: Mindfulness-based intervention
MDIS: Mood disorders insight scale
SCID: Structured clinical interview for DSM-IV disorders
